# A Memory of Early Life Physical Activity Is Retained in Bone Marrow of Male Rats Fed a High-Fat Diet

**DOI:** 10.3389/fphys.2017.00476

**Published:** 2017-07-07

**Authors:** Dharani M. Sontam, Mark H. Vickers, Elwyn C. Firth, Justin M. O'Sullivan

**Affiliations:** ^1^The Liggins Institute, University of Auckland Auckland, New Zealand; ^2^Gravida: National Centre for Growth and Development, University of Auckland Auckland, New Zealand; ^3^Department of Sport and Exercise Science, University of Auckland Auckland, New Zealand

**Keywords:** exercise, bone marrow, gene expression, memory

## Abstract

Studies have reported opposing effects of high-fat (HF) diet and mechanical stimulation on lineage commitment of the bone marrow stem cells. Yet, how bone marrow modulates its gene expression in response to the combined effects of mechanical loading and a HF diet has not been addressed. We investigated whether early-life (before onset of sexual maturity at 6 weeks of age) voluntary physical activity can modulate the effects of a HF diet on male Sprague Dawley rats. In the bone marrow, early-life HF diet resulted in adipocyte hypertrophy and a pro-inflammatory and pro-adipogenic gene expression profile. The bone marrow of the rats that undertook wheel exercise while on a HF diet retained a memory of the early-life exercise. This memory lasted at least 60 days after the cessation of the voluntary exercise. Our results are consistent with the marrow adipose tissue having a unique response to HF feeding in the presence or absence of exercise.

## Introduction

Childhood obesity has reached epidemic levels worldwide (WHO, 2016). The metabolic dysfunction induced by obesity in childhood persists into adulthood (Mattsson et al., 2008; Juonala et al., 2011; Schmidt et al., 2011; Lloyd et al., 2012). There is a genetic component to this complex phenotype. However, environmental factors, including excess energy intake and reduced physical activity, make a measurable contribution to the increasing obesity prevalence. As such, physical activity has long been considered as a non-pharmacological strategy to combat obesity and its associated co-morbidities (Tuomilehto et al., 2001; Misra et al., 2008).

Achieving optimal bone mass and strength during growth has biologically relevant effects on skeletal competence in both early and later life (Baxter-Jones et al., 2011). Childhood and adolescence are periods of rapid bone growth and attaining optimal bone mass during early life reduces the prevalence of orthopedic diseases such as fracture during childhood, adolescence, and later life (Ducher and Bass, 2007; Rizzoli et al., 2010). The opinion that obesity has a protective effect on bone due to the increased mechanical demand imposed by the excessive body weight on the skeleton has been challenged in recent times (Goulding et al., 2001, 2008; Skaggs et al., 2001; Sabhaney et al., 2014). Evidence from animal studies heavily favors the hypothesis that obesity may be detrimental to bone health during growth with several studies reporting an inverse relationship between excess adiposity, and the mechanical and micro-structural properties of bone (Cao et al., 2009; Woo et al., 2009; Lorincz et al., 2010; Ionova-Martin et al., 2011; Zhao, 2013; Yan et al., 2015). Understanding the effects of excessive adiposity on the growing skeleton is essential given the rising rates of childhood obesity.

Mechanical loading in the form of physical activity and/or exercise has the ability to influence body composition and bone properties. Physical activity not only suppresses the accumulation of fat mass through increased energy expenditure, but also promotes an anti-inflammatory environment, thereby helping to reduce chronic low-grade inflammation associated with excess adiposity (Gleeson et al., 2011). In the bone marrow, the adipocytes and osteoblasts share a common mesenchymal progenitor whose lineage commitment can be influenced by environmental factors (Beresford et al., 1992; Parhami et al., 2001; David et al., 2007). Studies have reported opposing effects of high-fat (HF) diet and mechanical stimulation on the lineage commitment of bone marrow stem cells. For example, an atherogenic HF diet reduced the ability of marrow stromal cells to differentiate into osteoblasts in a female mouse model (Parhami et al., 1999). By contrast, the application of high frequency-low magnitude mechanical stimuli in mice resulted in a marrow environment that favored osteogenesis (Luu et al., 2009).

Skeletal loading causes changes in the expression of genes involved in adipocyte and osteoblast differentiation and function (David et al., 2007; Menuki et al., 2008). Early studies on environmental impacts on bone and/or marrow gene expression have addressed the effects of HF diet or exercise independently (Xiao et al., 2010; Lange et al., 2013; Sontam et al., 2015a). Yet, how mechanical loading in the presence of a HF diet modulates bone marrow gene expression has not been addressed. As such, it is unclear if the beneficial effects of physical activity are retained or lost following cessation of an exercise program (Järvinen et al., 2003; Yasari et al., 2007; Sertie et al., 2013). In the present study, we determined the changes in bone marrow gene expression that were associated with early-life (before onset of sexual maturity at 6 weeks of age; Sengupta, 2013) HF diet accompanied by voluntary physical activity. In male Sprague-Dawley (SD) rats, we measured the effects of HF diet (45% Kcal from fat) and voluntary wheel running, before and after puberty, on body composition, bone mass indices, and gene expression in marrow isolated from the femur. We identified significant changes in gene expression and physiological measures. The gene expression changes were retained but did not prevent rebound obesity in previously exercised animals. Our results are consistent with the bone marrow having a unique and long-lasting response to HF feeding, depending on the presence or absence of exercise.

## Materials and methods

### Study design

This study was approved by and carried out in accordance with the recommendations of the University of Auckland Animal Ethics Committee (AEC001432). Eighty male weanling rats (aged 22 days) born from normal Sprague-Dawley (SD) dams were allocated at weaning (controlling for body weight and litter of origin) into either: (A) a chow-fed group (C-SED; *n* = 20) which obtained 18% of total calories from fat (Diet 2018, Teklad Global 18% Protein Rodent Diet, Harlan Teklad, USA) and was allowed spontaneous movement within standard cages; or (B) a HF-fed group (*n* = 60) which obtained 45% of total energy intake from fat (D12451, Research Diets, USA). The HF-fed group was further divided into three sub-groups: (1) a HF sedentary (HF-SED, *n* = 20) which had only spontaneous movement within the cage for the duraticon of the experiment; (2) HF late exercise (HF-LEX, *n* = 20) group which was allowed spontaneous cage activity up to D_67_ and access to a running wheel from D_67_ to D_120_; and (3) a HF early exercise group (HF-EEX, *n* = 20) which had access to a running wheel from D_22_ to D_60_ (Model 80859, Lafayette Instrument, Lafayette, IN, USA), and only spontaneous movement for the remainder of the experiment. All rats were housed as pairs throughout the study to avoid the stressors associated with singleton housing (Lin and Scott, 2011). All animals had *ad libitum* access to food and water and were housed in a temperature controlled room (25°C) with a 12 h light/dark cycle. Food and water intake was recorded at regular intervals.

“Early-life” is regarded as the period before onset of sexual maturity, which is around 6 weeks of age; sexual maturity is some 3 weeks before onset of early adulthood (end of the 8th week of life; Sengupta, 2013). The EEX period was begun at weaning as physical activity before puberty results in greater and more persistent bone morphology responses than if exercise begins after puberty. Finally, the exercise periods differed (i.e., EEX, 38 days; LEX—53 days) to ensure the period of lack of exercise after cessation of EEX lasted firmly into adulthood in order to test for a memory.

Wheel exercise data were recorded at 15 min intervals using dedicated monitoring software (Model 86065). Previous studies by our group (Sontam et al., 2015b) and others (Holy and Zérath, 2000) observed a distinct circadian pattern in the wheel activity of young rats. Preliminary analyses of the exercise data confirmed that the rats in the HF-EEX group used the wheel preferentially during the dark period (18:00–6:00 h) with minimal activity during the day. For this reason, only dark period wheel exercise data were analyzed.

Animals were fasted overnight, anesthetized using sodium pentobarbitone (60 mg/kg, IP) and culled by decapitation. Five pairs of rats from each group were culled at either D_60_ or D_120_.

### Body composition

Dual energy x-ray absorptiometry (DXA), using dedicated small animal software (Lunar Hologic, GE, Waltham, MA, USA), was performed on animals under light isofluorane anesthesia to determine the body composition of animals in the week preceding each cull.

### Bone and marrow collection

A sterile saw was used to partially saw the left femur at the junction of: (1) the distal and middle thirds; and (2) the middle and proximal thirds. The femur was then snapped to obtain the mid-diaphysis. Bone marrow was isolated from the diaphyseal bone by centrifugation (10,000 rpm, 4°C, 30 s), and immediately snap-frozen in liquid nitrogen and stored (−80°C) until analysis.

### Calculation of bone marrow adipocyte area

Measurements of adipocyte area were performed on femur tissue that had been prepared for histology, to test for evidence of between-group differences in marrow response. This was not a primary objective or hypothesis of the study and the *post-hoc* nature of the measurements meant that we were unable to measure total adiposity as we did not have the whole bone available to us. Thus, we were able to quantify adipocytic size in the metaphysis just inferior (distal) to the site of RNA_seq_ sampling. Briefly, a Metasystems VSlide scanner (MetaSystems, Version 2.1.124) running Metafer4 (version 3.9.2) and coupled with MetaXpress (Molecular Devices, Version 5.3.0.4) was used to image toluidine blue-stained sections already processed to study joint cartilages. After threshold-based segmentation of the region immediately distal to the metaphyseal trabecular bone, images (x20 magnification) were stitched using the VSlide software, and viewed using Image Viewer (VSViewer, MetaSystems, Version 2.0). “Snapshot”.tiff images were processed in ImageJ calibrated to convert pixels into distance (μm), thresholded (range 228–255), and binarised for adipocyte identification. Using the “wand” tool within the ROI manager, 10 adipocytes at random were selected, and the area of each was measured.

### Peripheral quantitative computed tomography (pQCT)

The length of the right femur was measured (sliding caliper) from the most distal aspect of the lateral femoral condyle to the proximal extent of the major trochanter. The bone was lodged in a plastic tube filled with saline, taking care that no air bubbles were present, and scanned (XCT Research SA+ pQCT machine, StratecMedizinTechnik, Pforzheim, Germany). The reference line was positioned at the distal aspect of the condyles in the scout view before the machine made one scan at 50 and 19% of the femoral length, at the diaphysis and metaphysis respectively, voxel size 70 μm. Outcome measures were chosen to enable determination of cortical bone density and bone architecture, as previously described (Gasser, 2012).

### RNA extraction

Bone marrow total RNA was extracted using a modification of Ayturk et al. (2013). Briefly, bone marrow was ground into a fine powder using a mortar and pestle cooled in liquid nitrogen. The frozen bone marrow powder was transferred to a sterile microcentrifuge tube containing 1 mL TRIzol® (#15596-026, Life Technologies, Carlsbad, CA, USA), mixed thoroughly by shaking, homogenized by sonication (Bandelin Sonopuls HD2070, Bandelin, Berlin Germany), and extracted using TRIzol®-chloroform extraction. Total RNA was purified using an RNeasy Mini Kit (#74104, Qiagen, Hilden, Germany) and on-column DNA digestion was performed to remove traces of genomic DNA (RNase-free DNase Set, #79254, Qiagen, Hilden, Germany). RNA quantity was measured using a Qubit™ 3.0 fluorometer with the RNA High Sensitivity Assay Kit (#Q32855, ThermoFisher Scientific, Waltham, MA, USA). RNA quality was assessed using an Agilent Bioanalyser (Model 2100; Agilent Technologies, Santa Clara, CA, USA). RNA integrity numbers (RIN) for the samples ranged from 6.1 to 8.0 (Supplementary Table 3).

### RNA-sequencing

RNA samples were sequenced using an Illumina Hi-seq 4000 (Supplementary Table 3; 150 bp paired end; Novogene, Beijing, China). For shipping, purified total RNA samples were mixed with RNAstable® in a 96-well plate (#90220-001, Biomatrica, San Diego, CA, USA) and dried according to manufacturer's instructions.

### Sequence read processing, alignment, and differential gene expression analysis

Sequencing reads were trimmed (phred score < 30 discarded) using PRINSEQ (http://prinseq.sourceforge.net/). Processed reads that were <50 bp in length were discarded before further analyses.

Processed reads were aligned to the rat reference genome (NCBI version Rnor_5.0) using TopHat (version 2.1.0) (Supplementary Table 1; Trapnell et al., 2009). Gene model annotations were provided in a GTF file. Differential gene expression was determined using Cuffdiff (Cufflinks version 2.2.1) (Trapnell et al., 2010). Ten biological replicates from HF-SED, HF-EEX and HF-LEX conditions and seven biological replicates from the C-SED condition were sequenced and used for differential gene expression analysis.

### Pathway analysis

Pathway analysis was performed using the Ingenuity pathway analysis (IPA, Ingenuity Systems Inc., Redwood City, CA, USA) software package. Data was uploaded into IPA with the ingenuity knowledge base as the reference set and a *P* < 0.05.

IPA was used to identify the biological functions, physiological processes and diseases associated with the differentially expressed genes. IPA downstream effects analysis was used to identify biological functions that were predicted to be up- or down-regulated based on the observed gene expression changes. An “activation *z*-score” of ≥2 or ≤ −2 was taken as significant for the biological functions that were predicted to be affected by the treatment (Ingenuity Systems, 2011). A regulatory effects analysis was performed to identify potential upstream regulators that explain the observed gene expression changes in the dataset.

### Statistical analyses

Statistical analyses were performed using SigmaPlot 13.0 (SysStat Software Inc., CA, USA). One-way analysis of variance (ANOVA) was used to identify statistically significant differences between the groups. The Holm–Sidak method was used to correct for multiple comparison testing. Where the data failed the equal variance test, ANOVA on Ranks followed by the Tukey test for multiple comparisons was used to determine the between-group differences.

## Results

### Effect of HF diet and early exercise on growth, caloric intake, and body composition at day 60

Caloric intake was significantly increased overall in the HF-fed groups compared to chow-fed controls and was not affected by exercise (Supplementary Figure 1). Similarly, body weight gain until day 60 was significantly increased in all HF-fed groups when compared to chow-fed controls (Supplementary Figure 2).

**Figure 1:**
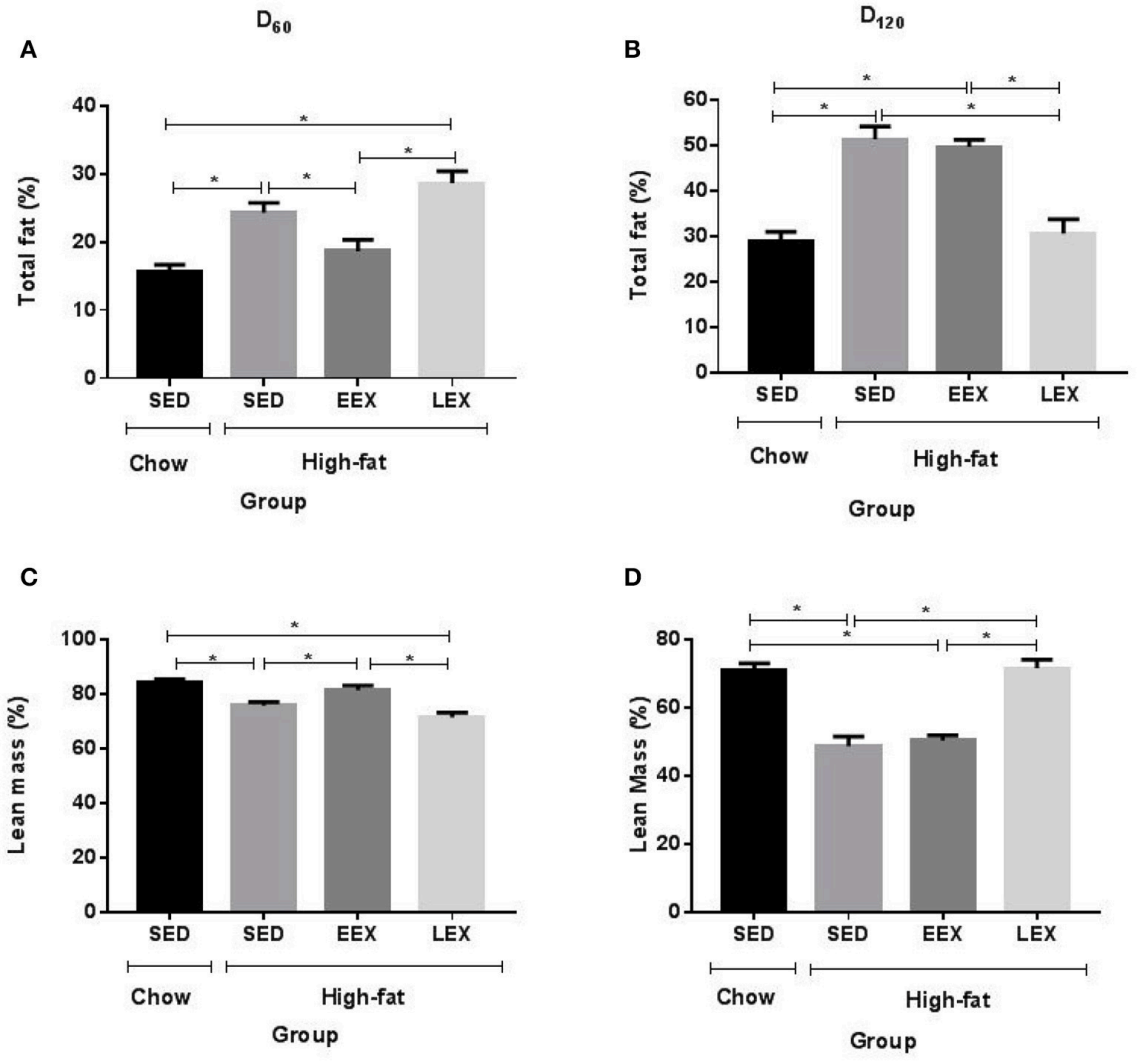
Total fat and lean mass percentage is affected by current but not previous physical activity. **(A)** Total fat percentage at D_60_ **(B)** Total fat percentage D_120_ **(C)** Lean mass percentage at D_60_ **(D)** Lean mass percentage at D_120_. ^*^P < 0.05. All parameters were analyzed using one-way ANOVA followed by Holm–Sidak method for multiple comparison. SED, sedentary; EEX, early-exercise; LEX, late-exercise.

The HF-SED and HF-LEX groups were significantly heavier than C-SED, whereas the HF-EEX group was not different from C-SED (Table 1). Similarly at day 60 both the total body fat (%) (Figure 1A) and fat:lean ratio were higher in the HF-SED and HF-LEX groups compared to C-SED (Table 1). The total body fat and fat:lean ratio was not different between the C-SED and HF-EEX groups. Lean mass (%) (Figure 1C) was lower in HF-SED and HF-LEX groups than in C-SED and HF-EEX groups.

**Table 1:** Rat body composition at D60 and D120.

**Parameter**	**Age**	**C-SED**	**HF-SED**	**HF-EEX**	**HF-LEX**
Body weight (g)	D_60_	289.90 ± 5.98^b^^,^^d^	320.70 ± 6.28^a^^,^^d^	305.80 ± 10.21^d^	361 ± 9.05^a^^,^^b^^,^^c^
	D_120_	560.6 ± 17.3^b^^,^^c^	704 ± 25.82^a^^,^^d^	688.10 ± 22.71^a^^,^^d^	528.20 ± 16.81^b^^,^^c^
Total fat %	D60	15.67 ± 0.97^b^^,^^d^	24.32 ± 1.42^a^^,^^c^	18.65 ± 1.68^b^^,^^d^	28.65 ± 1.75^a^^,^^c^
	D_120_	28.93 ± 1.99^b^^,^^c^	51.32 ± 2.86^a^^,^^d^	49.67 ± 1.56^a^^,^^d^	30.59 ± 3.14^b^^,^^c^
Fat:Lean	D_60_	0.19 ± 0.01^b^	0.33 ± 0.03^a^	0.23 ± 0.03^d^	0.41 ± 0.04^a^^,^^c^
	D_120_	0.42 ± 0.04^b^^,^^c^	1.12 ± 0.12^a^^,^^d^	1 ± 0.06^a^^,^^d^	0.47 ± 0.08^b^^,^^c^
Lean mass%	D_60_	84.37 ± 0.98^b^^,^^d^	75.73 ± 1.41^a^^,^^c^	81.45 ± 1.70^b^^,^^d^	71.43 ± 1.76^a^^,^^c^
	D_120_	70.97 ± 1.97^b^^,^^c^	48.7 ± 2.87^a^^,^^d^	50.36 ± 1.56^a^^,^^d^	69.41 ± 3.14^b^^,^^c^
BMC/BW	D_60_	0.0186 ± 0.00^b^^,^^c^^,^^d^	0.0200 ± 0.00^a^	0.0197 ± 0.00^a^	0.0199 ± 0.00^a^
	D_120_	0.0239 ± 0.00^c^^,^^d^	0.0251 ± 0.00	0.0265 ± 0.00^a^	0.0259 ± 0.00^a^
BMD (g/cm^2^)	D_60_	0.11 ± 0.00^b^^,^^c^^,^^d^	0.12 ± 0.00^a^^,^^d^	0.12 ± 0.00^a^^,^^d^	0.13 ± 0.00^a^^,^^b^^,^^c^
	D_120_	0.17 ± 0.00^b^^,^^c^	0.18 ± 0.00^a^	0.19 ± 0.00^a^^,^^d^	0.17 ± 0.00^c^

*Values are mean ± S.E.M. P < 0.05 compared to*

a *C-SED;*

b *HF-SED;*

c *HF-EEX;*

d *HF-LEX. Groups: C-SED, Chow + sedentary; HF-SED, High-fat diet + sedentary; HF-EEX, High-fat diet+ early exercise; HF-LEX, High-fat diet + late exercise. Statistical analyses were performed using one-way ANOVA. The Holm–Sidak method was used to correct for multiple comparisons*.

### Effect of HF diet and early/late exercise on growth, caloric intake, and body composition at day 120

Both the HF-EEX and HF-LEX groups were fed a HF diet ad-libitum from weaning until day 120. The HF-EEX group was allowed running wheel activity from day 23, and there was a gradual increase in distance completed until day 60 (from 988.59 ± 347.05 m cage^−1^ night^−1^ increasing to 9971.97 ± 1933.34 m cage^−1^ night^−1^), at which point the running wheels were removed (Supplementary Figure 3). The HF-LEX group had wheel access between days 67 and 120. Similar to the HF-EEX animals, the HF-LEX animals exhibited a gradual increase in distance covered (from 1377 ± 254 m cage^−1^ night^−1^ increasing to a mean daily distance of 9907 ± 2550 m cage^−1^ night^−1^), followed by a gradual decrease toward the end of the study period (to 3254 ± 1169 m cage^−1^ night^−1^). As expected most activity (~98%) occurred during the dark phase of the light cycle.

Following the cessation of exercise, linear body growth in the HF-EEX group returned to match that of the animals in the HF-SED group (Supplementary Figure 2). Animals undergoing the late voluntary exercise protocol exhibited a significant decrease in body weights compared to animals in the HF-SED and HF-EEX groups, such that the HF-LEX animals were not different from those in the C-SED group by day 120. Accordingly, final body weights at day 120 were significantly higher for animals in the HF-SED and HF-EEX groups compared to those of the C-SED and HF-LEX groups (Table 1). This was reflected in a similar pattern of total body fat (%) (Figure 1B) and fat:lean ratios (Table 1) which were significantly increased in animals from the HF-SED and HF-EEX groups when compared to those from the C-SED and HF-LEX groups. Conversely, relative lean mass (%) was significantly decreased in HF-SED and HF-EEX groups compared to C-SED and HF-LEX groups (Figure 1D).

### Bone marrow adipocyte area

**Figure 2:**
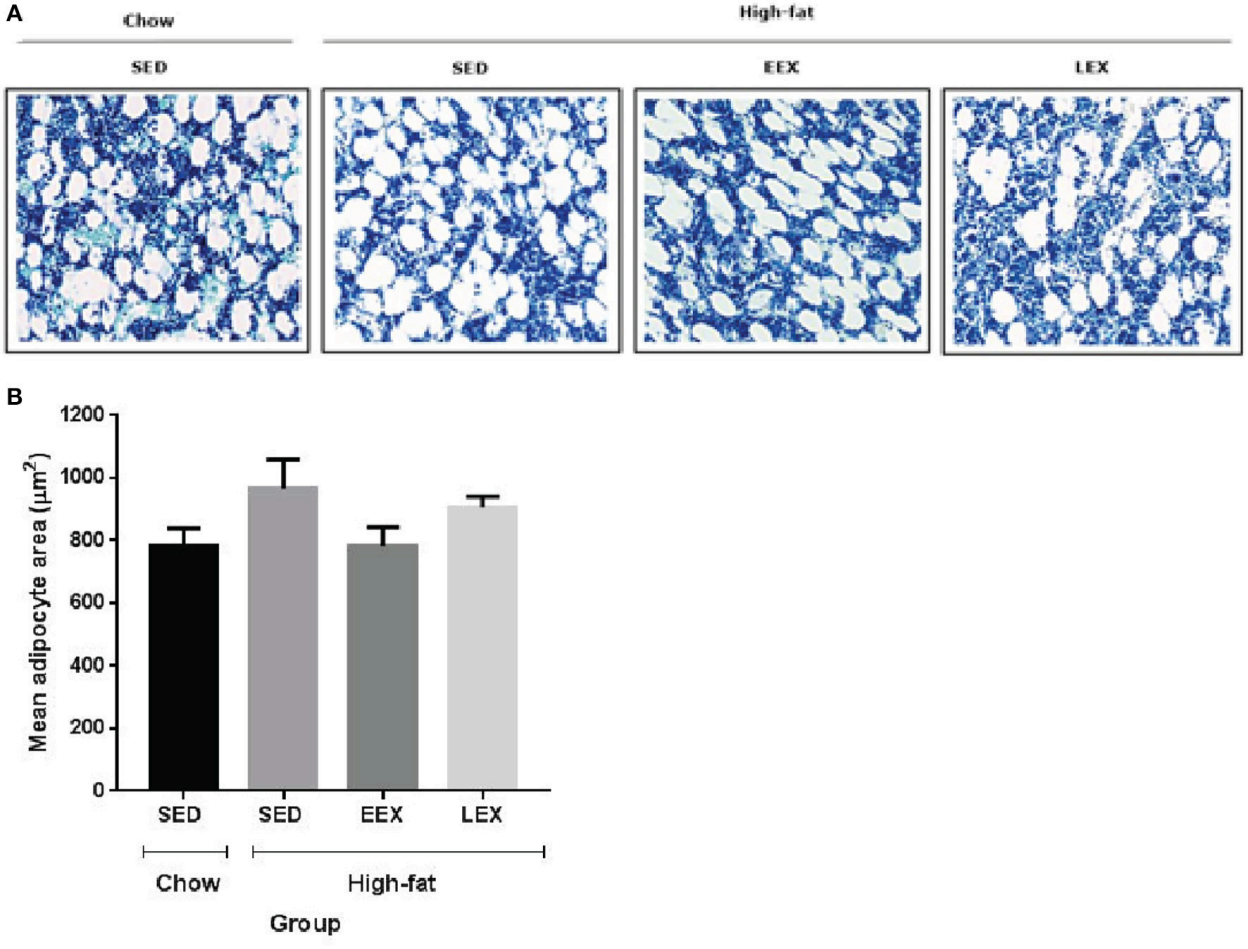
Mean marrow adipocyte area of the experimental groups at D_120_. **(A)** Representative images of bone marrow adipocytes in the experimental groups. **(B)** Graphical representation of mean adipocyte area. There were no statistically significant differences between any of the experimental groups with respect to their bone marrow adipocyte area. However, the mean marrow adipocyte area of the HF-SED group was larger than C-SED, HF-EEX, and HF-LEX groups.

At D_120_, animals in the HF-SED group had the largest adipocyte area (965.76 ± 93.04 μm^2^). Animals in the C-SED group had the smallest adipocyte area (782.86 ± 54.89 μm^2^), while the adipocyte area for animals in the HF-EEX group (783.03 ± 60.07 μm^2^) was almost identical to that in the C-SED group. By contrast, the adipocyte area in animals in the HF-LEX exercise group (906.74 ± 34.01 μm^2^) was more similar to that in the HF-SED animals (Figure 2). The difference in adipocyte area between the HF-EEX and HF-SED groups approached statistical significance (*p* = 0.054).

### pQCT-measured bone parameters

There were no differences in total area, periosteal circumference or endosteal circumference within the mid-diaphysis from the femurs of any of the groups (Table 2). By contrast, animals from all of the HF-fed groups (i.e., HF-SED, HF-EEX, and HF-LEX) had greater cortical thickness and volumetric bone mineral density (_v_BMD) than animals from the C-SED group (Table 2). Similarly, animals from the HF-SED and HF-EEX groups had significantly higher cortical bone mineral content (BMC) than animals from the C-SED group (Table 2). Only animals from the HF-EEX group had significantly increased cortical bone area than animals from the C-SED group.

**Table 2:** Femoral cortical and trabecular bone properties in the experimental groups at the end of the late exercise period.

	**C-SED**	**HF-SED**	**HF-EEX**	**HF-LEX**
**DIAPHYSIS**				
Total area^§^	15.28 ± 0.57	15.53 ± 0.53	16.58 ± 0.27	15.52 ± 0.47
Cortical BMC^¥^	11.51 ± 0.35^b^^,^^c^	12.93 ± 0.26^a^	13.45 ± 0.29^a^	12.46 ± 0.34
Cortical _v_BMD^‡^	1371.42 ± 3.34^b^^,^^c^^,^^d^	1397.67 ± 5.14^a^	1391.53 ± 3.78^a^	1392.17 ± 4.73^a^
Cortical area^§^	8.40 ± 0.25^c^	9.26 ± 0.21	9.66 ± 0.20^a^	8.95 ± 0.25
Cortical thickness^‡^	0.73 ± 0.01^b^^,^^c^^,^^d^	0.81 ± 0.01^a^	0.81 ± 0.01^a^	0.78 ± 0.01^a^
Periosteal circum^‡^	13.84 ± 0.25	13.95 ± 0.23	14.43 ± 0.12	13.95 ± 0.21
Endosteal circum^‡^	9.28 ± 0.22	8.85 ± 0.24	9.32 ± 0.07	9.07 ± 0.19
**METAPHYSIS**				
Total BMC^¥^	18.33 ± 2.02	18.4 ± 1.81	19.34 ± 1.92	19.34 ± 1.92
Total _v_BMD^‡^	591.52 ± 63.71^b^^,^^c^^,^^d^	547.48 ± 52.59^a^	530.50 ± 51.5^a^	530.50 ± 51.50^a^
Total area^§^	31.10 ± 3.47^c^	33.61 ± 3.28	36.48 ± 3.58^a^	33.66 ± 3.27
Trabecular area^§^	16.50 ± 2.04^b^^,^^c^	19.54 ± 1.93^a^	22.15 ± 2.24^a^^,^^d^	19.04 ± 1.92^c^
Trabecular BMC^¥^	6.03 ± 0.74^c^	6.05 ± 0.63^c^	7.32 ± 0.75^a^^,^^b^	6.29 ± 0.64
Trabecular _v_BMD^‡^	366.21 ± 38.96^b^^,^^c^^,^^d^	308.97 ± 30.26^a^	330.06 ± 31.74^a^	331.69 ± 33.46^a^

*Results are mean ± SEM. Groups: C-SED, Chow + sedentary; HF-SED, High-fat diet + sedentary; HF-EEX, High-fat diet + early exercise; HF-LEX, High-fat diet + late exercise. Statistical analyses were performed using one-way ANOVA and Holm–Sidak method for multiple comparison. Superscript letter in any column shows P < 0.05 significance with respectively vs*.

a *C-SED;*

b *HF-SED;*

c *HF-EEX;*

d *HF-LEX. BMC, bone mineral content; _v_BMD, volumetric bone mineral density. Circum., circumference;*

§ *, mm^2^;*

¥ *, mg;*

*^‡^, mg/cm^3^; ^‡^, mm*.

In the metaphysis, there was no statistically significant difference in total BMC between any of the groups (Table 2). Total _v_BMD and trabecular _v_BMD of animals from all three HF-fed groups (i.e., HF-SED, HF-EEX, and HF-LEX) was significantly lower than that in animals from the C-SED group (Table 2). The trabecular bone area in animals from the HF-SED and HF-EEX groups was significantly larger than that from animals in the C-SED group, which latter was not different from those in the HF-LEX group. In addition, the trabecular area of animals from the HF-EEX group was greater than that in HF-LEX animals. In the HF-EEX group total area and trabecular BMC were significantly higher than observed for animals from the C-SED group, and trabecular BMC was higher than that in the HF-SED group also.

### The bone marrow gene expression profile of the HF-SED group was pro-adipogenic and pro-inflammatory

RNA-seq identified 328 genes that were up-regulated and 210 genes that were down-regulated in the HF-SED group when compared to the C-SED group. Ingenuity Pathway Analysis (IPA) revealed that genes involved in inflammation, metabolism, and connective tissue disorders were significantly enriched within the differentially expressed genes in bone marrow from animals in the HF-SED group (Table 3). Downstream IPA effects analysis found that “quantity of adipose tissue” function was positively activated, based on the direction of changes of the genes in the dataset (*z*-score: +1.815; Supplementary Table 1). Genes belonging to the ontology category “morphology of bone” were also identified as significantly over-represented in the set of differentially expressed genes (Supplementary Table 1).

**Table 3:** Biological functions, physiological processes, and diseases that were over-represented by genes that were differentially regulated due to the high-fat diet.

	***P*-value range**	**N° of genes**
**PHYSIOLOGICAL SYSTEM DEVELOPMENT AND FUNCTION**		
Hematological system development and function	5.12E^−04^–2.88E^−19^	164
Immune cell trafficking	5.12E^−04^–2.88E^−19^	102
Tissue morphology	4.63E^−04^–3.02E^−19^	136
Lymphoid tissue structure and function	4.55E^−04^–1.81E^−14^	104
Humoral immune response	3.88E^−04^–2.82E^−14^	62
**DISEASES AND BIOLOGICAL FUNCTION**		
Inflammatory response	5.13E^−04^–8.25E^−14^	161
Immunological disease	5.23E^−04^–3.10E^−12^	121
Metabolic disease	4.63E^−04^–4.62E^−12^	89
Connective tissue disorders	4.63E^−04^–9.29E^−12^	87
Inflammatory disease	5.13E^−04^–9.29E^−12^	108

**Figure 3:**
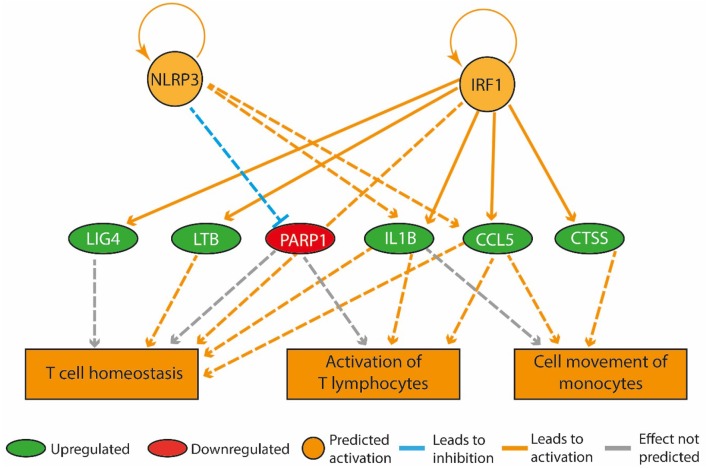
A high-fat diet in early life upregulated genes that promote inflammation in the bone marrow. The regulatory network pictured consists of three tiers. The top tier consists of predicted upstream regulators that might explain the gene expression changes observed in the experiment. NLRP3 and IRF1 are the upstream regulators whose predicted activated state may explain the expression changes in LIG4, LTB, PARP1, IL1B, CCL5, and CTSS (middle tier). The observed upregulation of LIG4, LTB, IL1B, CCL5, and CTSS and the downregulation of PARP1 leads to a predicted increase in T cell homeostasis, activation of T lymphocytes and cell movement of monocytes (bottom tier).

The expression of genes coding for actin alpha 1 (*Acta1*), cell death inducing DFFA-like effector C (*Cidec*), perilipin 1 (*Plin1*), and phosphoenolpyruvate carboxykinase (*Pck1*) was up-regulated more than two-fold in the HF-SED group compared to the C-SED group. In contrast to previous reports of an inverse relationship between adiposity and *Adipoq* mRNA levels, we observed higher *Adipoq* gene expression in the HF-SED group compared to the C-SED group (Supplementary Table 1). Among the most significant regulatory networks we identified by IPA, four were associated with inflammatory pathways (Table 3). “Activation of T-lymphocytes,” “T cell homeostasis,” and “cell movement of monocytes” were all predicted to be upregulated in the bone marrow of HF-SED rats (Figure 3). In addition to their roles in inflammation, LTB, IL-1β, and CCL5 all have been shown to participate in bone metabolism (Garrett et al., 1987; Thomson et al., 1987; Boyce et al., 1989; Yano et al., 2005; Wintges et al., 2013).

### Voluntary physical activity later in life promoted an anti-inflammatory environment in bone marrow

**Figure 4:**
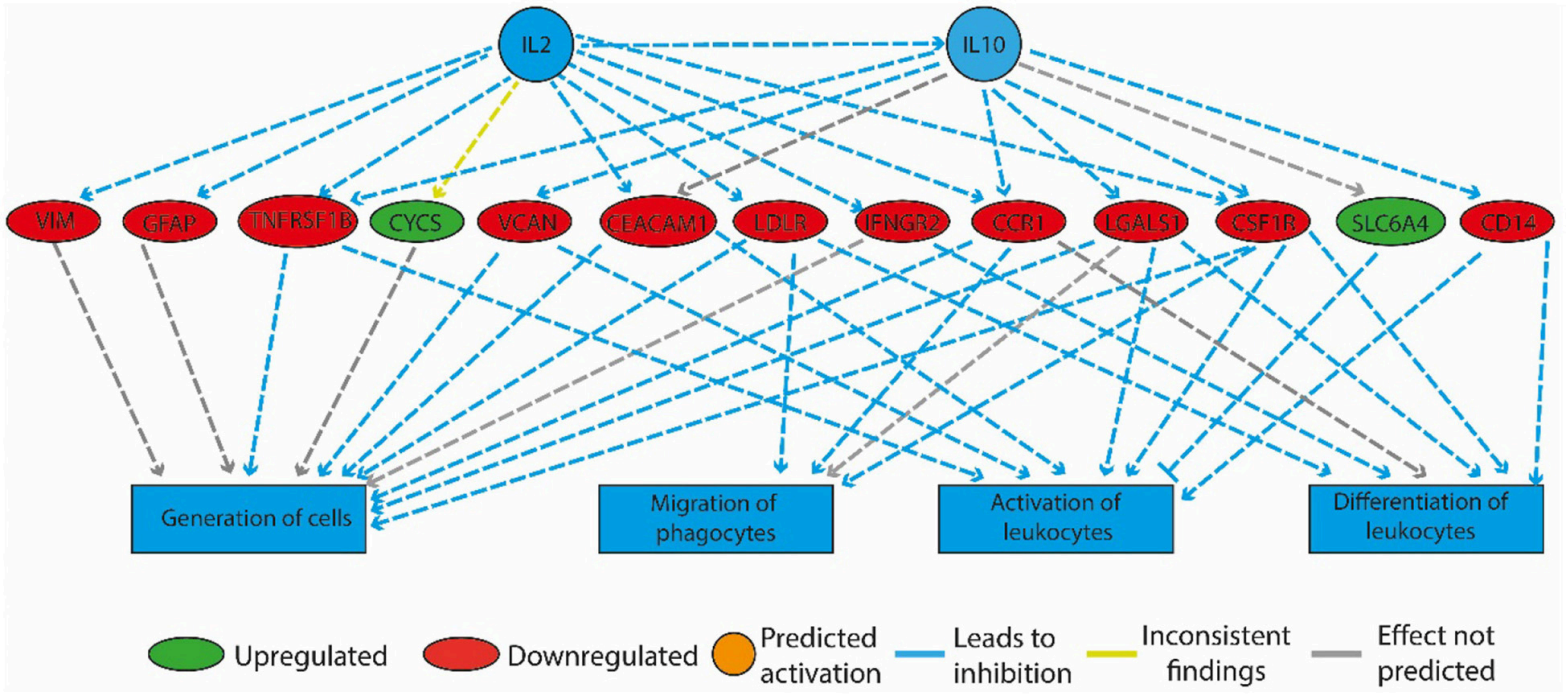
Physical activity in high-fat fed rats downregulated genes involved in inflammation. The regulatory network that is illustrated consists of three tiers. The top tier consists of predicted upstream regulators that might explain the gene expression changes observed in the experiment. IL2 and IL10 are the upstream regulators whose predicted activated state may explain the altered regulation of the middle tier genes. The observed gene expression changes lead to a predicted decrease in “generation of cells,” “migration of phagocytes,” “activation,” and “differentiation of leukocytes” (bottom tier).

In the HF-LEX group, voluntary physical activity between D_67_ and D_120_ combined with lower food intake induced changes in the D_120_ bone marrow gene expression. In the D_120_ bone marrow samples 179 genes were differentially expressed from HF-LEX animals when compared to those from HF-SED, with 76 genes upregulated due to the exercise, and 102 genes down-regulated. Among the five most significantly affected biological functions and diseases identified by IPA, three were related to inflammation, and related ontologies (immunological disease, inflammatory response and inflammatory disease; Table 4). As observed in the HF-EEX group (Table 3), the most significantly affected regulatory networks also belonged to the “inflammation” category. Notably, most genes in the IL2, IL10 inflammatory regulatory network were down-regulated in the HF-LEX group compared to HF-SED group (Figure 4). Thus, exercise reduced the expression of genes that were involved in inflammation within HF-LEX animals while HF-SED animals exhibited up-regulation of these pro-inflammatory genes.

**Table 4:** Biological functions, physiological processes, and diseases that were over-represented by genes that were differentially expressed in HF-LEX group compared to HF-SED group.

	***P*-value range**	**N° of genes**
**PHYSIOLOGICAL SYSTEM DEVELOPMENT AND FUNCTION**		
Immune cell trafficking	6.36E^−03^–6.37E^−12^	42
Hematological system development and function	6.36E^−03^–3.50E^−11^	55
Organismal functions	2.72E^−03^–2.65E^−07^	9
Cardiovascular system development and function	6.07E^−03^–7.00E^−07^	37
Connective tissue development and function	5.74E^−03^–7.25E^−06^	30
**DISEASES AND BIOLOGICAL FUNCTION**		
Immunological disease	4.70E^−03^–4.58E^−13^	50
Inflammatory response	6.36E^−03^–1.07E^−09^	65
Connective tissue disorders	5.94E^−03^–1.75E^−09^	34
Inflammatory disease	4.70E^−03^–1.75E^−09^	42
Skeletal and muscular disorders	6.47E^−03^–1.75E^−09^	48

Genes with known roles in osteoclast formation and function were down-regulated in the HF-LEX animals. RANKL-mediated induction of osteoclastogenesis requires a co-stimulatory immunoreceptor tyrosine-based activation motif (ITAM) pathway which is activated in response to ligation of osteoclast-associated receptors such as OSCAR. Moreover, in a pro-inflammatory environment, the complement C3 and C5 bind to their receptors (C3aR and C5aR) on osteoblasts and promote the expression of RANKL (Ignatius et al., 2011). We observed a down-regulation of *C5aR1* and *Oscar* transcripts in the HF-LEX group. Notably, neuropilin 1 (NRP1), which acts with semaphorin 3A (SEMA3A) to inhibit RANKL-mediated activation of osteoclasts, was up-regulated in the HF-LEX group (Hayashi et al., 2012).

### Bone marrow gene expression retained a memory of early-life exercise

**Figure 5:**
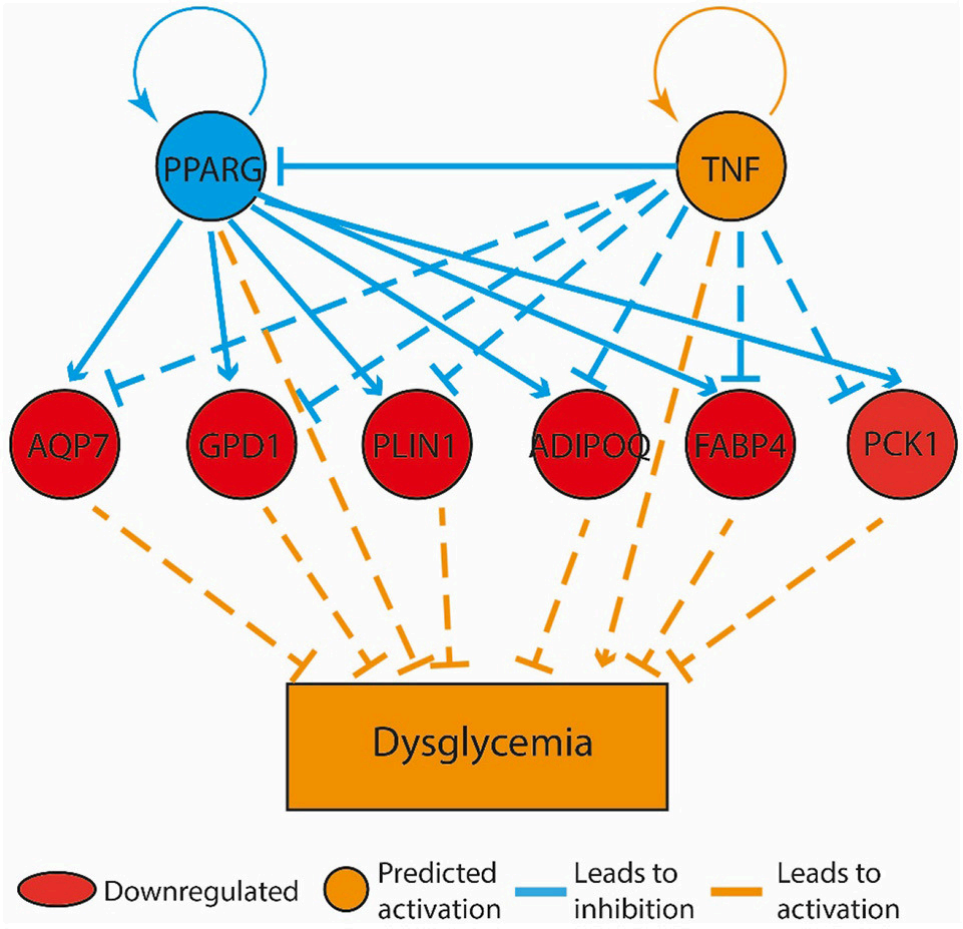
Bone marrow gene expression retained a memory of physical activity following 60 days of physical activity cessation. The top tier consists of predicted upstream regulators that might explain the gene expression changes observed in the experiment. PPARG and TNF are the upstream regulators whose predicted states may explain the altered regulation of the middle tier genes. The observed gene expression changes lead to a predicted increase in “dysglycemia” (bottom tier).

Compared to the HF-SED group, 128 genes were differentially regulated (48 up- and 80 down-regulated) in the HF-EEX group, of which 14 genes showed two-fold or greater up-regulation while 16 genes showed two-fold or greater down-regulation (Supplementary Table 2). Genes with roles in adipogenesis and adipocyte function were down-regulated, and formed the only regulatory network that was significantly differently expressed between the HF-EEX and HF-SED conditions (Figure 5).

We also observed differential expression of genes with roles in both adipocyte and bone metabolism. Crucially, we observed significant down-regulation of *Cdo1* (log ratio: −0.87) and *Dlk1* (log ratio: −0.47) in the bone marrow of the HF-EEX compared to HF-SED group. Our observations thus indicate that the early-life exercise in the presence of a HF diet influenced gene expression significantly such that at D_120_, the gene expression profile remained different from that in animals only exposed to a HF diet (HF-SED).

## Discussion

How bone marrow modulates gene expression in response to diet and exercise is not known. In this study, the HF diet resulted in an obese phenotype by D_60_ consistent with previous observations (Zhao, 2013; Doucette et al., 2015; Reynolds et al., 2015). Continued exposure to the HF diet, through until D_120_ of life, was associated with changes in bone micro-architecture and bone marrow gene expression, including up-regulation of adipocyte and inflammatory genes, in adult male rats. Voluntary physical activity early in life was sufficient to cause significant changes in body composition in HF-fed rats at D_60_ such that the total fat percentage, lean mass percentage and fat:lean ratio resembled that of animals on the control diet. At D_120_, measures of bone microarchitecture and gene expression identified a pattern of gene transcription (i.e., a “transcriptional memory”) of the early-life exercise that lasted at least 60 days after cessation of wheel running activity. However, the transcriptional memory of early-life exercise did not prevent rebound to the obese phenotype, by D_120_, in the absence of continuing voluntary wheel running exercise.

Transcription levels for genes involved in mature adipocyte function and lipid metabolism (*Adipoq, Cidec, Pck1*, and *Plin1*) were increased in bone marrow of sedentary animals exposed to a HF diet. Both *Plin1* and *Cidec* encode proteins that are lipid droplet-associated and predominantly expressed in adipose tissue (Konige et al., 2014) control many aspects of lipid metabolism including lipid droplet size, triglyceride storage, and lipolysis (Sun et al., 2013; Konige et al., 2014). Up-regulation of *Cidec* gene expression in response to a HF diet (60% of total energy from fat) has been previously reported within epididymal adipose tissue of wild-type C57B6 mice (Reynolds et al., 2015).

Bone marrow transcript levels from the *Adipoq* gene showed up-regulation in response to the HF diet, in contrast to previous reports of *Adipoq* gene expression decreasing in perigonadal white adipose tissue and epididymal fat pads in mice fed HF diets (Barnea et al., 2006; Bullen et al., 2006). This apparent contradiction may be due to differences in morphology and cellular composition of the different tissues (Trubowitz and Bathija, 1977; Bathija et al., 1979; Griffith et al., 2009), consistent with *Adipoq* mRNA and protein levels being significantly greater in bone marrow adipose than white adipose tissue depots in mice subjected to caloric restriction (Cawthorn et al., 2014). Therefore, we conclude that there are bone marrow adipose tissue-specific changes to *Adipoq* transcript levels in response to our HF diet.

Hypercaloric-induced obesity is associated with chronic low-grade inflammation, characterized by increased pro-inflammatory cytokine production (Gregor and Hotamisligil, 2011). Consistent with this, we observed a pro-inflammatory gene expression profile that included up-regulation of *Il-1*β, *Ltb*, and *Ccl5* transcript levels within bone marrow of rats fed the HF diet. These inflammatory cytokines have roles in bone metabolism, and IL-1β has been shown to stimulate bone resorption (Nguyen et al., 1991; Pfeilschifter et al., 2009) in response to a HF diet (Shu et al., 2015). Similarly, LTB acts synergistically with TNF-α and IL-1β and is a potent stimulator of bone resorption (Stashenko et al., 1987). The observed increases in IL-1β, LTB and other pro-resorptive cytokines (e.g., TNF-α and RANKL) are consistent with the HF diet increasing bone resorption in our animals. However, further confirmation of this linkage requires empirical testing.

In response to the HF diet there were significant increases in cortical BMC, cortical _v_BMD, and trabecular _v_BMD, consistent with other reports that showed a positive association between HF diet and bone mass indices (Ma et al., 2010; Lecka-Czernik et al., 2015). These most likely resulted from increased mechanical loading or androgenic factors, while other reports indicate an inhibitory effect of HF diet associated with effects on the differences in bone formation and resorption (Lorincz et al., 2010; Cao and Picklo, 2015; Shu et al., 2015). There were no exercise-associated between-group differences in cortical bone mass parameters in the HF-fed animals. Critically, trabecular bone mass was significantly higher in the HF-EEX group than in either of the non-exercised groups.

Although, there were no significant phenotypic differences in the bone micro-architecture as a result of this post-puberty (D_67−120_) exercise in the HF animals, compared to the HF-SED group, this physical activity combined with lower caloric intake was associated with down-regulation of genes involved in inflammation, and of genes that promote osteoclastogenesis (i.e., *Csf1r, Ccr1*, and *C5aR1*). Transcript levels for *Nrp1*, which is osteoprotective through its enhancing effect on osteoblast differentiation, were upregulated. As such, post-puberty voluntary wheel running and lower caloric intake led to gene expression changes consistent with a reduction in the production of osteoclasts, which resorb bone, and an increase in osteoblasts, which produce it.

Voluntary pre-pubertal (D_23−60_) exercise affected the total fat and lean mass percentages in animals on a HF diet such that they were more similar to a control group on a standard chow diet. However, within 60 days of ceasing voluntary exercise, there was a return to an obese phenotype such that their total fat and lean mass percentages were not significantly different from sedentary animals fed a HF diet. This is consistent with the principle of training reversibility whereby the physiological adaptations induced by physical training are partially or completely lost when the training is stopped or markedly reduced (Mujika and Padilla, 2000). Similar observations of training reversibility have been observed previously in SD and Wistar rats on HF diets (Yasari et al., 2007; Sertié et al., 2015).

Pre-pubertal exercise was associated with significant differential transcript levels for 128 genes when compared to HF-fed sedentary animals. The difference is notable, as these two groups of animals received identical diets and exercise regimes between D_67_ and D_120_, and gene expression changes in responses begin hours not days or weeks after novel physical activity begins. This is consistent with a “programmed” and long-lived memory of the early-life voluntary exercise.

The mechanism of the memory remains to be determined and could be due to: (1) sustained changes to the cellular composition of the bone marrow; or (2) epigenetic changes that are affecting the program of gene expression. These mechanisms are not mutually exclusive and it is likely that the memory of the physical stimuli is retained through a combination of both compositional and epigenetic changes (Ntanasis-Stathopoulos et al., 2013; Marędziak et al., 2015). For example, reductions in bone marrow fat and increased numbers of mesenchymal stem cells have been identified in 4-week-old mice trained on a treadmill (Marędziak et al., 2015). Moreover, bone marrow mesenchymal stem cells undergo changes in their DNA methylation when stimulated by mechanical signals (Arnsdorf et al., 2010). While the transcript levels of the mouse *Adipoq* gene are linked to the obesity-dependent methylation status of its promoter (Kim et al., 2015), the ability of mechanical stimulation to influence the epigenetic profile of *Adipoq* and subsequent changes in gene expression has not been established. Future investigations should focus on the mechanisms through which *Aqp7, Gpd1, Plin1, Adipoq, Fabp4*, and *Pck1* gene expression is modulated in response to mechanical stimulation, and for how long after cessation such modulation persists. Such studies will enable the elucidation of the mechanism(s) that lead to a memory of early-life exercise, which appears to offer possibilities to inhibit expression of some undesirable effects of HF diets on homeostasis in the longer term. Were such suppression preserved for even longer periods, it may be possible to attenuate or prevent some aspects of the early life programming that increase health risks in adulthood.

This study was designed to determine if early-life mild voluntary activity, as opposed to imposed moderate to high (and possibly aversive and/or stressful) physical activity, would have lasting effects on tissues, cells and genes. We used only male rats, because they are less active than females. Using both sexes would have increased inter-group variance in at least some outcome measures, due to sex-dependant differences in development of bone (Wang et al., 2003) and many other normal physiological processes ranging from neurodevelopment (Galea et al., 2013) feeding behavior (Fukushima et al., 2004) voluntary activity (Rosenfeld, 2017) to exercise-induced physiological cardiac hypertrophy (Foryst-Ludwig and Kintscher, 2013). Resource constraint is likely why especially initial studies cannot be conducted in both sexes concurrently, despite this obviously being desirable (Krizo and Mintz, 2015). Rats were housed as pairs, to optimize animal well-being in conformance with approval procedures, and this limited the correlation of individual rats' activity with other outcome measures. Also, we assumed spontaneous cage activity was similar across the five cages within a particular group at a particular age, since we lacked facilities to measure such activity, and this also limited ability to compare spontaneous cage activity with wheel activity. Now that we have shown a significant wheel exercise effect, further work is required to characterize the exercise undertaken in detail.

An unanticipated confounder in the current study was the reduced caloric intake in the HF-diet fed group that had access to voluntary exercise between D_67_ and D_120_. The combined effect of the reduction in caloric intake and exercise could be associated with the similarities in body weight, total fat, and lean mass percentages of the HF-LEX and chow-fed groups. However, the HF-fed late exercise group had greater whole body BMC (Rosenfeld, 2017), cortical and trabecular _*v*_BMD, cortical thickness and total metaphyseal _*v*_BMD than was observed in the chow-fed animals.

The biological significance of the study lies in the significant differences in gene expression patterns in regulatory pathways important in the understanding of obesity and its many effects. Gene expression of particular regulatory pathways was influenced by the early-life mild voluntary exercise, which was expected, but the persistence of their exercise-induced gene expression pattern into adulthood was not. The phenomenon indicates that some key aspects of the obese phenotype may be suppressed and others not. For instance, the whole body composition phenotype reverted to control values after exercise ceased, redolent of recent reports of the metabolically healthy obese phenotype (Roberson et al., 2014). This contrasts with the generally poor success of activity strategies to combat existing obesity (Marchesini et al., 2016). The nature of the world-wide obesity epidemic demands exploration of alternative strategies to reduce morbidity and soaring healthcare costs (Hammond and Levine, 2010), and assurance of early life activity is likely to be one with high chances of positive effects, even if limited to particular aspects of phenotype.

The possible translational significance in terms of human health is that our observations confirm previous data of early exercise effects in some rodent studies. The exercise was voluntary and of mild to moderate intensity. We avoided imposition of more intensive exercise since this would hardly be acceptable in strategies designed for encouraging musculo-skeletal activity in infants and young children, especially those born small for gestational age or from obese pregnancy, since such groups are predisposed to obesity and/or less than optimal bone development (Taylor and Poston, 2007; Chen et al., 2010). The opportunity for altering gene expression through increased musculoskeletal activity may be highly time (age)-sensitive within infancy and childhood, because gene and phenotype plasticity decline. Lack of detailed knowledge of when and how various levels of physical activity at young ages may affect regulation of gene expression is probably why results of exercise studies in young rodents are conflicting. The translational opportunity lies in determining which gene regulatory pathways can be effected by early life physical activity, and the specification of the physical activity regimens needed to elicit persistent effects. We conclude that some regulatory pathways related to preserving or restoring homeostasis in energy metabolism can be retained, even though whole body phenotype changes induced by such mild exercise were not retained after early-life exercise ceased. Aspects of the observed memory of early life exercise are supportive of both positive and negative consequences for later life disorders. Therefore, while there is no impact on rebound adiposity, we contend that the altered immune responses we observed will impact on future risks of inflammation-associated disease. Such impacts need to be empirically confirmed.

## Data availability

All sequencing data has been deposited in Gene expression omnibus (GSE97376).

## Author contributions

DS: Investigation, writing-original draft preparation; MV, EF, JO: Conceptualization. MV, EF, and JO: writing- review and editing; MV, EF, JO: Supervision.

### Conflict of interest statement

The authors declare that the research was conducted in the absence of any commercial or financial relationships that could be construed as a potential conflict of interest.
