# Supplementary material for: A Memory of Early Life Physical Activity Is Retained in Bone Marrow of Male Rats Fed a High-Fat Diet
Source: Front Physiol. 2017 Jul 7;8:476. doi: 10.3389/fphys.2017.00476 (PMC5500658; doi:10.3389/fphys.2017.00476)
Supplement: Supplementary file 1 [file Table1.PDF]

Supplementary Figures

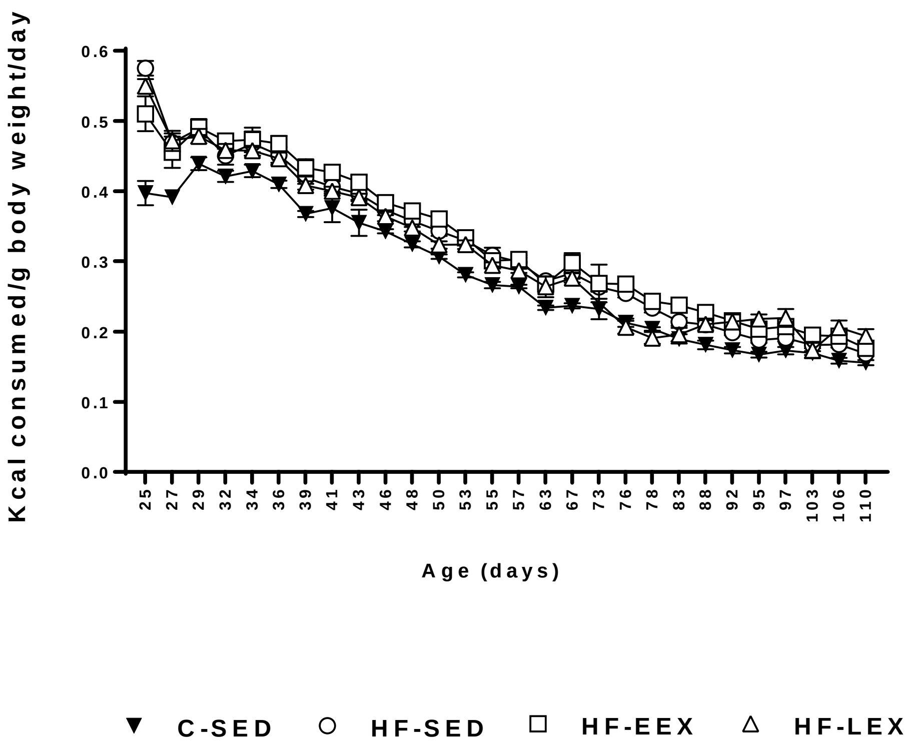

Fig S1: Food consumption during the experimental period. Kcal consumed per gram of body weight was calculated by dividing the number of calories consumed per pair of rats per day by the average body weight of the rats. C-SED = Control+sedentary; HF-SED = High-fat diet+sedentary; HF-EEX = High-fat+early-exercise; HF-LEX = High-fat+late-exercise.
