# Supplementary material for: A Memory of Early Life Physical Activity Is Retained in Bone Marrow of Male Rats Fed a High-Fat Diet
Source: Front Physiol. 2017 Jul 7;8:476. doi: 10.3389/fphys.2017.00476 (PMC5500658; doi:10.3389/fphys.2017.00476)
Supplement: Supplementary file 2 [file Table2.PDF]

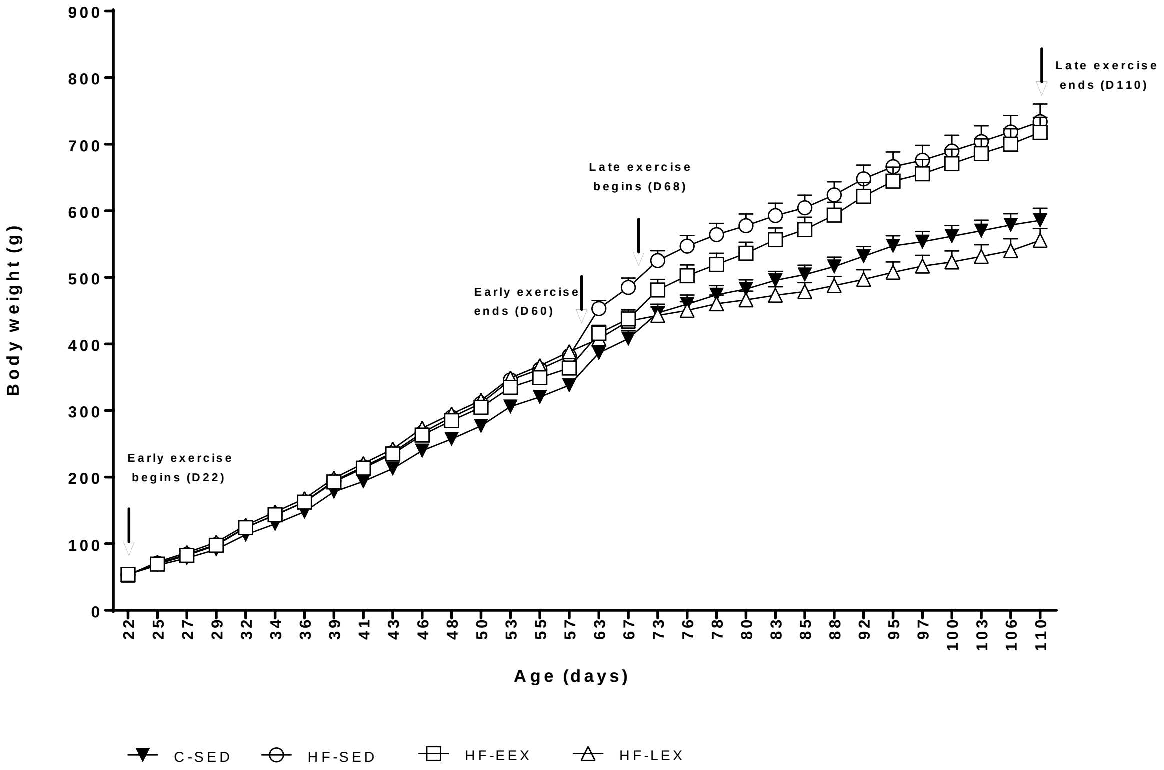

Fig S2: Body weights of the experimental groups. Body weights (g) were measured and recorded. C-SED: Chow+sedentary; HF-SED: High-fat+sedentary; HF-EEX: High-fat+early-exercise; HF-LEX: High-fat+late-exercise. All data are presented as means ± S.E.M with an n=10 per group unless otherwise stated.
