# Supplementary material for: A Memory of Early Life Physical Activity Is Retained in Bone Marrow of Male Rats Fed a High-Fat Diet
Source: Front Physiol. 2017 Jul 7;8:476. doi: 10.3389/fphys.2017.00476 (PMC5500658; doi:10.3389/fphys.2017.00476)
Supplement: Supplementary file 3 [file Table3.PDF]

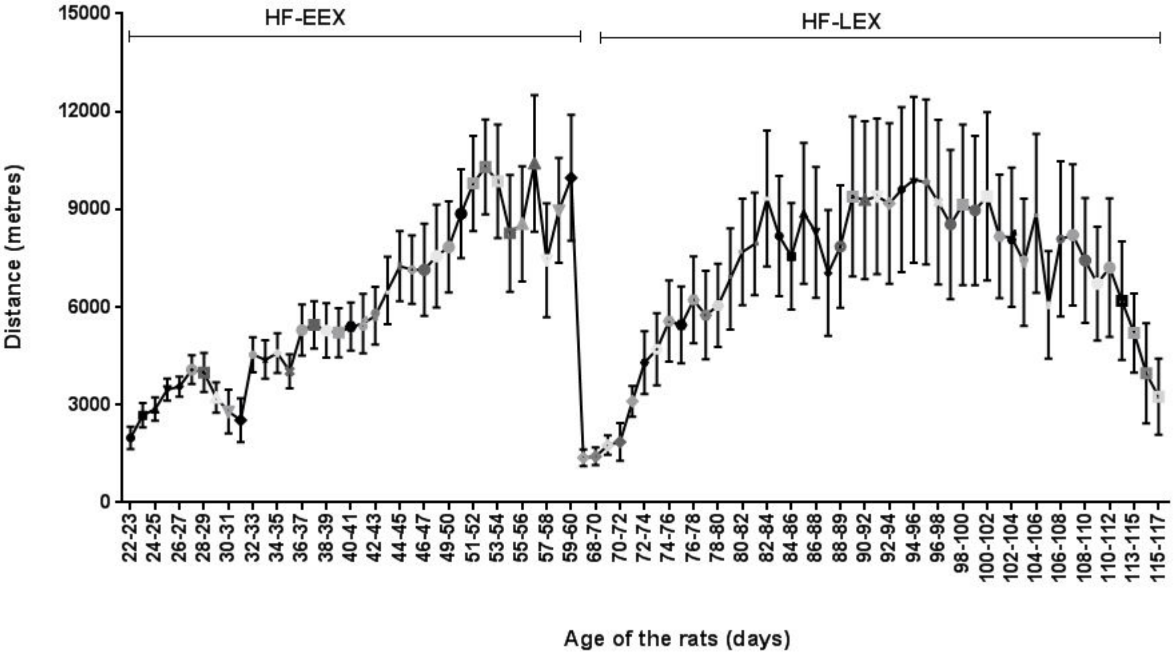

Fig S3: Wheel activity of HF-EEX and HF-LEX groups. At the beginning of the early exercise period, the mean distance ran by rats in the HF-EEX group during the dark period was 1988.59 ±347.05 m $cage^{-1}$ $night^{-1}$ increasing to 9971.97 ±1933.34 m $cage^{-1}$ $night^{-1}$ at the end of the early exercise intervention. At the beginning of the late exercise period (D_{67}), the animals ran an average daily distance of 1377 ± 254 m $cage^{-1}$ $night^{-1}$ increasing to a mean daily distance of 9907 ± 2550 m $cage^{-1}$ $night^{-1}$ on D_{94-96}. The mean daily distance run then gradually declined to 3254 ± 1169 m $cage^{-1}$ $night^{-1}$ at the end of the exercise period (D_{115}-D_{120}). HF-EEX: High-fat+early-exercise; HG-LEX: High-fat+late-exercise
