# Supplementary material for: A Memory of Early Life Physical Activity Is Retained in Bone Marrow of Male Rats Fed a High-Fat Diet
Source: Front Physiol. 2017 Jul 7;8:476. doi: 10.3389/fphys.2017.00476 (PMC5500658; doi:10.3389/fphys.2017.00476)
Supplement: Supplementary file 4 [file Image1.PDF]

Table S1: Genes with functions in 'quantity of adipose tissue' and 'morphology of bone' were significantly enriched among the differentially expressed genes in HF-SED group compared to the C-SED group.

**Biological function**	**Gene ID**	**Experimental log ratio**
**Quantity of adipose tissue**	*Acta1*	4.25
	Adipoq	1.435
	Angptl4	0.36
	Apoe	0.289
	Arntl	0.557
	Cebpa	0.371
	Cidec	1.553
	Cxcl14	0.286
	Dlk1	0.719
	Hdc	0.293
	Hrh3	0.419
	Hspa5	-0.359
	Lpl	0.452
	Pck1	1.914
	Plin1	1.506
	Plin2	0.397
	Plvap	0.391
	Ucp3	0.692
**Morphology of bone**	*Adipoq*	1.435
	Apoe	0.289
	Arntl	0.557
	Btd	0.627
	Ccr1	0.559
	Cdo1	0.876
	Col1a1	-0.352
	Col1a2	-0.354
	Ddr1	0.688
	Dlk1	0.719
	Eln	0.441
	Evc2	-1.548
	Fos	-0.648
	H19	3.053

	Hdc	0.293
	Ibsp	-0.413
	Itgb3	-0.546
	Kif3a	-0.536
	Klf2	0.603
	Lfng	0.294
	Lmo4	0.289
	Mgp	0.48
	Mmp13	-0.71
	Ndrg2	0.41
	P2ry6	0.761
	Pax5	0.413
	Plxnd1	0.274
	Ptprc	0.309
	Slc35d1	-0.456
	Smad6	0.814
	Sox4	0.39
	Tfeb	0.442
	Thbs1	-0.533
	Tyrobp	0.288
