# Supplementary material for: A Memory of Early Life Physical Activity Is Retained in Bone Marrow of Male Rats Fed a High-Fat Diet
Source: Front Physiol. 2017 Jul 7;8:476. doi: 10.3389/fphys.2017.00476 (PMC5500658; doi:10.3389/fphys.2017.00476)
Supplement: Supplementary file 5 [file Image2.PDF]

Table S2: Genes that were differentially expressed greater than or equal to two fold in HF-EEX group compared to HF-SED group

	**Gene ID**	**Experimental log ratio**	**FDR adjusted P-value**
**Up-regulated genes**	*RT1-Ba*	4.91205	0.0034366
	LOC257642	2.76297	0.0034366
	LOC102553290	2.63363	0.0034366
	LOC102553223	2.0183	0.0034366
	Lyc2	1.52106	0.0034366
	Acta1	1.48393	0.0034366
	Rmrp	1.44246	0.0034366
	LOC102555237	1.4395	0.0034366
	LOC310926	1.32289	0.0034366
	LOC691846	1.21214	0.0416151
	Mboat2	1.03156	0.0085999
	Ifit1	1.01612	0.0034366
**Down-regulated genes**	*Calml3*	-1.05604	0.0034366
	Aqp7	-1.09151	0.0316926
	LOC100359922	-1.10714	0.0034366
	Mcpt8l3	-1.13039	0.0034366
	LOC100911529	-1.18141	0.0381043
	Nnat	-1.18203	0.0034366
	Atp6v1e2	-1.22248	0.0229127

	Plin1	-1.2978	0.0034366
	Cidec	-1.30112	0.0034366
	S100b	-1.31347	0.0034366
	LOC100364500	-1.4387	0.0034366
	Car3	-1.47486	0.0034366
	Adipoq	-1.58064	0.0034366
	Pck1	-1.58224	0.0034366
	Thrsp	-1.61622	0.0034366
	Pcp4l1	-1.75507	0.0034366
