# Supplementary material for: A Memory of Early Life Physical Activity Is Retained in Bone Marrow of Male Rats Fed a High-Fat Diet
Source: Front Physiol. 2017 Jul 7;8:476. doi: 10.3389/fphys.2017.00476 (PMC5500658; doi:10.3389/fphys.2017.00476)
Supplement: Supplementary file 6 [file Image3.PDF]

Table S3: RNA quality and alignment summary of RNA-seq reads to the rat transcriptome

Group	Rat ID	RIN	Total read pairs	Aligned pairs	Concordant pair alignment rate (%)	Multiple alignments (%)	Discordant alignments (%)
**C-SED**	15	6.7	16400160	12810745	77.5	5.7	0.8
	63	7.4	20460740	16141151	78.0	5.7	1.2
	64	6.1	15181769	11974157	78.3	5.3	0.8
	72	7	15831916	12487443	77.9	5.4	1.3
	79	7.5	14986215	11786369	78.2	7.2	0.6
	7	6.5	14338747	10739900	73.8	5.8	1.5
	8	7.6	12765046	9865364	75.8	5.9	1.9
**HF-SED**	29	7.3	18040968	14970936	82.6	6.4	0.5
	30	7.5	18422550	15214910	81.9	5.4	0.8
	45	7.3	18670642	15378292	82.0	5.6	0.5
	46	7.2	17610594	14513939	82.0	6.0	0.5
	61	7.5	15608673	13063035	83.4	6.2	0.3
	62	6.7	15187945	12248170	80.3	4.9	0.4
	37	7.4	13591531	11106307	81.3	5.8	0.5
	38	7.2	18130925	14889895	81.6	5.0	0.7
	53	7.8	14588952	11473040	78.0	6.2	0.8
	54	7.4	15036020	11677801	76.5	5.6	1.6
**HF-EEX**	9	7.5	14168861	12795752	80.0	5.4	0.5
	10	7.4	15620760	12522086	79.7	5.9	0.6
	49	7.1	17080289	13826557	80.6	6.5	0.5
	50	7.5	21754907	17633701	80.7	6.1	0.5
	33	6.6	14106232	11267763	79.5	4.7	0.5
	34	7.1	20328275	16263921	79.6	6.2	0.5
	25	7.1	16126943	12738016	78.5	5.6	0.6
	26	7.5	17324954	13802004	79.3	5.9	0.5
	65	7.3	14388493	11457989	79.1	5.5	0.7
	66	7.7	14104123	11284043	79.6	5.8	0.5
**HF-LEX**	3	6.9	12458551	10042081	80.2	6.1	0.5
	4	7.4	13404783	10785675	80.1	5.6	0.5
	43	7	18249588	14977785	81.7	5.4	0.5
	44	7.5	16701631	13770993	82.0	5.9	0.5
	67	7.5	18406507	15196744	82.2	6.0	0.5
	68	6.9	17946935	14872165	82.5	5.3	0.4
	51	7.3	17056287	13952239	81.0	5.4	0.9
	52	7.2	16857159	13849895	81.8	5.7	0.4
	75	7.2	20113702	16658376	82.4	5.8	0.5
	76	7.4	16514911	13588472	81.5	6.2	0.9

Groups: C-SED, Chow + sedentary; HF-SED,High-fat diet + sedentary; HF-EEX, High-fat diet+ early-exercise; HF-LEX, High-fat diet + late-exercise.
